# The effect of delivery ball and warm shower on the childbirth experience of nulliparous women: a randomized controlled clinical trial

**DOI:** 10.1186/s13063-022-06358-x

**Published:** 2022-05-12

**Authors:** Parvaneh Sharifipour, Masoomeh Kheirkhah, Mojgan Rajati, Hamid Haghani

**Affiliations:** 1 Department of Reproductive Health and Midwifery, School of Nursing and Midwifery, Iran University of Medical Sciences, Tehran, Iran; 2 Nursing Care Research Center (NCRC), Department of Reproductive Health and Midwifery, School of Nursing and Midwifery, Iran University of Medical Sciences, Shahid Rashid Yasemi St., Vanak Square, Tehran, 1996713883 Iran; 3 Obstetricians Research Center, Motazedi Hospital, EMCKUMS, Keamanshah, Iran; 4 Department of Biostatistics, Iran University of Medical Sciences, Tehran, Iran

**Keywords:** Delivery ball, Warm shower, Midwife-centered care, Childbirth experience, Primiparous

## Abstract

**Introduction:**

Childbirth is a unique experience that affects women’s life. Midwives can play an effective role in creating positive birth experiences for women using non-pharmacological and supportive methods. Accordingly, this study aims to determine the effect of delivery balls and warm showers on childbirth experiences of primiparous women.

**Methods:**

This clinical trial was conducted on primiparous pregnant women who referred to the Motazedi Hospital in Kermanshah, Iran. Sampling was done from eligible individuals by a continuous method, and pregnant women were assigned to the three groups of delivery balls plus warm showers or A (*n* = 35), delivery balls or B (*n* = 35), and control or C (*n* = 35). The use of the ball at the dilation of 4 cm was similar in the two groups of A and B, but the first group used a warm shower at the dilatation of 7 cm as well. The control group also received routine delivery care. Besides, demographic information forms consisting of the pregnancy history and some information about the mother and her infant were completed. Additionally, childbirth experience questionnaires (CEQ) were completed by the women two hours after childbirth. The analysis of intervention effects was performed as per-protocol analysis.

**Results:**

There was a statistically significant difference in the mean score of the childbirth experience between the two groups of A and C (*p* = 0.001) after the intervention as well as between the groups of B and C (*p* = 0.001).

**Conclusion:**

The use of delivery balls and warm showers was effective in creating a positive childbirth experience. To create a positive childbirth experience in mothers, the use of both interventions (delivery balls and warm showers) is recommended.

**Trial registration:**

TCTR 20200408002. Prospectively registered on March 21, 2020.

## Introduction

Childbirth is one of the most memorable [1] and special life experiences in women [2]. Besides, as a common obstetric emergency [3], it is one of the most painful events women experience during their lifetime [2]. Some studies have reported that 33% of women have a negative childbirth experience [4]. Among the factors creating a negative childbirth experience in women, we can point to exposure anxiety [5], labor pain, and most importantly a sense of helplessness and lack of control during labor [6]. Negative childbirth experiences are associated with complications, such as higher willingness to undergo a cesarean section in next pregnancies and increased childbirth fear [7] miscarriage [8], post-traumatic stress disorder (PTSD), a lack of interpersonal relationships, poor mother-infant relationships, inappropriate use of mother-infant care services [9], and the sexual reconnection disorder [10].

The World Health Organization (WHO) defines a positive birth experience as the one meeting or even going beyond a woman’s previous expectations, including giving birth to a healthy infant in a safe environment and receiving continued practical support from kind and professionally qualified clinical staff [11]. Therefore, a midwife’s supportive role is one of the strong predictors of a woman’s perception of and satisfaction with her childbirth experience [12]. Midwives can reduce the severity of pain and anxiety in women during childbirth and create a positive experience of childbirth for them by providing a dedicated environment and maintaining privacy [13]; this can be realized using non-pharmacological and supportive methods and by improving psychological and emotional health among them [14].

One of the midwife-centered interventions during the labor process is to make a pregnant woman perform special movements using a delivery ball in different positions, especially in the vertical position [15, 16]. Given that most women prefer to have physiological childbirth, they can have a sense of success and personal control by doing such exercises. A warm shower is another midwife-centered intervention that creates a positive childbirth experience, which can be used in most hospitals. In addition, it is more welcomed by clients due to its simplicity and the high emotional support that it brings to women through their caregiver [17]. Such interventions reduce the need for epidural anesthesia, episiotomy, and instrumental delivery, with no side effects having been reported for the mother and her infant [18]. Comparison of the effects of a warm shower and intravenous injection of hyoscine on pleasant childbirth experiences shows the effectiveness of a warm shower [19], having also been introduced as an effective measure in reducing labor pain [20].

Such inexpensive and simple methods can be used to reduce and prevent side effects of pharmacological methods, including decreased consciousness and contractions as well as the decreased function of the respiratory system of the mother and her infant [21]; in addition, they can provide more comfort to women during childbirth and create a positive childbirth experience for them. Positive results are achieved through using non-pharmacological and midwife-centered interventions in helping women have normal physiological delivery during childbirth with minimal pain and complications, as well as positive childbirth experiences gained. However, this issue is still associated with many challenges in Iran in terms of awareness, acceptance, and implementation.

On the other hand, systematic review studies, meta-analyses, and clinical trials yield conflicting results concerning the effectiveness of interventions in terms of satisfaction with childbirth. In a study by Lathrap (2018), sitting in a hot tub was associated with the positive experience of childbirth. Similarly, in a study by Ulfsdottir (2019), sitting in a hot tub achieved a high score for the childbirth experience, yet it was not statistically significant. However, as far as we are concerned, no study has been conducted to compare the effects of the two methods of warm showers and delivery balls on the childbirth experience. Against this background, the present study was conducted to compare effects of delivery balls and those of warm showers on the childbirth experience among nulliparous women.

## Methods

### Design

This randomized clinical trial was conducted with three groups of A, B, and C. Data collection started from May 2020 to December 2020. In addition, this trial was funded by Iran University of Medical Sciences in April 2020 under research grant no. IR.IUMS.REC.1399.166. In addition, it was registered at the Thai Clinical Trials Registry (TCTR) Center under no. TCTR20200408002.The study population consisted of primiparous women who referred to the Motazedi Hospital affiliated to the Kermanshah University of Medical Sciences.

In group A, in addition to the routine labor care, labor pain control, fetal heart rate hearing, vaginal examination, fetal status examinations, fetal membranes, effacement, dilatation, the mother’s vital signs, and the like were considered. The first intervention was made at the dilation of 4 cm, in which special pelvic movements on the ball, in a sitting position, at a vertical angle of the legs, while moving back and forth, sides, up, and down, as well as the squatting position were simulated for an average of 30 min. Besides, at the dilation of 7 cm, the second intervention was made by taking a warm shower using a plastic cap. For the first 5 min, the women could wash their full body; in the next 15 min, they could wash any part of their body they wished.

In group B, in addition to the routine labor care, an intervention was made with the delivery ball, as in the first group. However, group C only received the routine labor care, labor pain control, fetal heart rate hearing, vaginal examination, fetal status examinations, fetal membranes, effacement, dilatation, the mother’s vital signs, and the like were considered. In addition, the personal and obstetric information form was initially completed by the co-researcher. Besides, the Birth Experience Questionnaire (CEQ) was completed by the women 2 h after the delivery.

### Participants and trial design

The inclusion criteria for the women included primiparous women, a gestational age of at least 37 weeks, a fetus with a single cephalic presentation, spontaneous onset of labor, cervical dilatation within the range of 4–5 cm, no contraindication for normal delivery, no high-risk pregnancy, no history of mental disorders according to the self-report, having minimum literacy, estimated fetal weight of less than 4000 g based on ultrasound and clinical examinations, and a normal pelvic diameter based on vaginal examinations. On the other side, the exclusion criteria included unwillingness to keep participating in the study and undergoing a cesarean section as an emergency.

To collect data, the researcher selected eligible individuals from among women who were in the active phase of labor through continuous sampling and briefed them on the study process. Next, the individuals who agreed to participate in the study were included. Besides, using the block randomization method at a 1:1:1 ratio (available at http://www.randomization.com), the eligible women were assigned into the two intervention and control groups by researcher. To determine the sequence of participant allocation based on the balanced block randomization method, it is necessary to know the total sample size, number of groups, and number of group iterations in each block (which were considered fixed). An epidemiologist, who was not part of the study, made a randomization list. For allocation concealment, the assignment list remained with the epidemiologist.

In the present study, the size of each block was considered equal to the number of the groups (3 groups per block). Due to the nature of the interventions in this trial, blinding was impossible. Next, a questionnaire completed by the participant after the study was presented by the co-researcher, as an outcome assessor, who was not aware of the study groups and their allocation. Additionally, statistical analysis was performed by a statistician who was not informed about the content provided to the study groups and their allocation.

### Assessment of trial variables

The variables of this study were measured by the following instruments:

#### Demographic and obstetric information form

This researcher-made questionnaire was designed in two parts. Accordingly, the first part was related to the women’s personal characteristics, including their age, education level, employment status, place of residence, economic status, and marriage duration. In addition, the second part was related to obstetric characteristics, including their number of pregnancies, number of abortions, gestational age, wanted pregnancies, and participation in maternity preparation classes. This part was completed by the co-researcher at the beginning of the study after asking questions from the samples.

#### Infant information form

This researcher-made questionnaire included neonatal information about fetal gender, weight, height, and the Apgar score at birth, which was completed by the principal researcher in the delivery room.

#### Childbirth Experience Questionnaire (CEQ)

The Childbirth Experience Questionnaire (CEQ), designed by Dencker (2010), consists of 22 items in 4 fields. In the Iranian version of the questionnaire, one item from the field of professional support has been removed, so it has 21 items in 4 fields of individual abilities (8 items), professional support (4 items), sense of security (6 items), and childbirth participation (3 items).

In addition, this questionnaire is based on a 4-point Likert scale, ranging from “it is not correct at all” (score 1), “it is somehow incorrect” (score 2), “it is somehow correct” (score 3), and “it is totally correct” (score 4). Scores in field of individual abilities range from 8 to 32; in the field of professional support, they range from 4 to 16; in the field of the sense of security, they range from 6 to 24; in the field of childbirth participation, they range from 3 to 12; and the total scores of childbirth experience range from 21 to 84, with higher score indicating a more positive experience. Reliability of this questionnaire has been confirmed with a Cronbach’s alpha of 0.70 [22].

In addition, Abbaspour et al translated this tool into Farsi. Furthermore, reliability of this tool was confirmed by the internal consistency method with a Cronbach’s alpha of 0.82 for the whole questionnaire, 0.71 for the field of individual abilities, 0.78 for the field of professional support, 0.69 for the field of the sense of security, and 0.58 for the field of participation [23]. This questionnaire was completed by the samples two hours after childbirth and was collected by the co-researcher.

### Sample size

The required sample size, at a 95% confidence level and 80% test power, and by assuming that the effect size of the intervention should be at least ES = 0.5 [20] to be considered statistically significant, was determined to be *n* = 32 in each group, using the following formula:

$$n=\frac{{\left({Z}_{1-{\alpha}\!\left/ \!2\right.}+{Z}_{1-\beta}\right)}^2}{ES^2}=\frac{{\left(1.96+0.84\right)}^2}{0.5^2}$$

In addition, upon taking into account the possibility of 10% sample dropout, the final sample size in each group was *n* = 35, and the total number of the samples was *n* = 105.

### Statistical analysis

For the inter-group comparison of the quantitative variables, including the age, duration of marriage, gestational age at delivery, and the infant’s weight/height, the one-way ANOVA was used. Besides, for the inter-group comparison of the qualitative variables, including the education level, place of residence, economic status, number of abortions, number of pregnancies, and participation in childbirth preparation classes, as well as regular reception of prenatal care and neonatal sex, a chi-square test was used. Besides, Fisher’s exact test was used for the inter-group comparison of the qualitative variables of pregnancy status, job, and the Apgar score of the first minute. Furthermore, the one-way ANOVA test was used to compare childbirth experiences, with the Tukey’s post hoc test used to compare childbirth experiences in pairs. Finally, data were analyzed by SPSS Statistics 21.0, and the significance level was considered less than 0.05. The analysis of intervention effects was performed as per-protocol analysis.

## Result

**Fig. 1:**
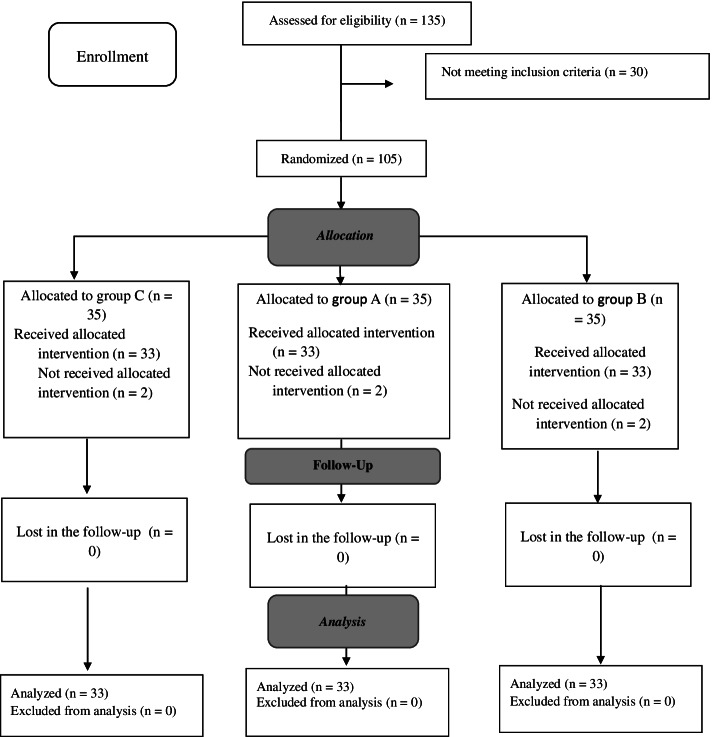
Allocation of the participants into the three study groups

A total of 135 women were evaluated for their eligibility to participate in the study. Accordingly, 105 eligible women (35 in group A, 35 in group B, and 35 in group C) were included in this study. Figure 1 shows the details of the women who were excluded from this study during the follow-up period and the final number of the women who were statistically analyzed.

There was no statistically significant difference among the three groups in terms of individual and pregnancy-associated variables, including the variables of age, level of education, employment status, place of residence, economic status, length of marriage, number of pregnancies, number of abortions, gestational age at childbirth, participation in childbirth preparation classes, receiving regular prenatal care, pregnancy status, neonatal gender, neonatal height and weight, and the Apgar score. In addition, the three groups were homogeneous (Table 1).

**Table 1:** Demographics and baseline characteristics of the study participants and results of comparison of the three study groups

Variables	Group A (***n*** = 33)	Group B (***n*** = 33)	Group C (***n*** = 33)	***p***-value
Women’s age (year) (*M* ± *SD*)	4.40 ± 24.27	4.9 ± 26.03	5.6 ± 24.88	**0.35**
Women’s education (%)				
Below high school diploma	13 (39.4)	7 (21.2)	14 (42.4)	**0.4**
High school diploma	14 (42.4)	19 (57.6)	13 (39.4)	
University	6 (18.2)	7 (21.2)	6 (18.2)	
Place of residence, *N* (%)				
City	23 (69.7)	27 (81.8)	22 (66.7)	**0.45**
Village	10 (30.3)	6 (18.2)	11 (33.3)	
Economic status (%)				
Good	7 (21.2)	7 (21.2)	3 (9.1)	**0.21**
Moderate	22 (66.7)	23 (69.7)	21 (63.6)	
Weak	4 (12.1)	3 (9.1)	9 (27.3)	
Women’s occupation (%)				
Housewife	30 (90.9)	31 (93.9)	31 (93.9)	**0.98**
Employed	3 (9.1)	2 (6.1)	2 (6.1)	
Duration of marriage (year), (*M* ± *SD*)	1.24 ± 2.39	1.47 ± 2.33	1.49 ±2.39	**0.98**
Gravida (%)				
1	27 (81.8)	26 (78.8)	27 (81.8)	**0.93**
2	6 (18.2)	7 (21.2)	6 (18.2)	
Number of abortions (%)				
0	27 (81.8)	26 (78.8)	27 (81.8)	**0.93**
1	6 (18.2)	7 (21.2)	6 (18.2)	
Gestational age at delivery (*X* ± *SD*)	0.97 ± 39.15	0.91 ± 39.03	0.85 ± 39.21	**0.71**
Participation in childbirth preparation classes (%)				
Yes	5 (15.2)	6 (18.2)	6 (18.2)	**0.99**
No	28 (84.8)	27 (81.8)	27 (81.8)	
Received regular prenatal care (%)				
Yes	30 (90.9)	30 (90.9)	29 (87.9)	**0.89**
No	3 (9.1)	3 (9.1)	4 (12.1)	
Pregnancy status (%)				
Wanted	31 (93.9)	30 (90.9)	26 (78.8)	**0.22**
Unwanted	2 (6.1)	3 (9.1)	7 (21.2)	
Baby’s gender (%)				
Female	16 (48.5)	19 (57.6)	18 (54.5)	**0.82**
Male	17 (51.5)	14 (42.4)	15 (45.5)	
Apgar score at the first minute (%)				
9–10	30 (90.9)	28 (84.8)	30 (90.9)	**0.78**
7–8	3 (9.1)	5 (15.2)	3 (9.1)	
Baby weight (*M* ± *SD*)	375 ± 3190	315 ± 3256	373 ± 3300	**0.45**
Baby height (*M* ± *SD*)	1.52 ± 50	2.35 ± 49.93	1.67 ± 50.36	**0.61**

However, there was a statistically significant difference in the mean scores of the childbirth experience after the intervention between groups A and C (*p* = 0.001) and between groups B and C (*p* = 0.001). In addition, there was a statistically significant difference in the mean scores of professional support between groups A and C (*p* = 0.02) and between groups B and C (*p* = 0.02). Furthermore, there was a statistically significant difference between the mean scores of the sense of security between group A and the control (*p* = 0.01). Additionally, there was a statistically significant difference in the mean scores of participation between groups A and C (*p* = 0.003) and between groups of B and C (*p* = 0.01) (Table 2).

**Table 2:** Numerical indicators of childbirth experience and its fields in the three groups

Group variable	Group A (***n*** = 33)	Group B (***n*** = 33)	Group C (***n*** = 33)	***p***-value (between groups)	** ***p*** -value (1–3)	** ***p*** -value (2–3)	** ***p*** -value (1-2)
Childbirth experience (*M* ± *SD*)	70.24 ± 6.95	67.8 ± 6.70	63.06 ± 7.07	0.001*	0.001	0.01	0.35
Childbirth experience (*X* ± *SD*)	17.56 ± 1.73	16.96 ± 1.67	15.76 ± 1.76	0.001*	0.001	0.01	0.35
Individual ability (*M* ± *SD*)	3.25 ± 0.39	3.15 ± 0.44	3.05 ± 0.4	0.14*			
Professional support (*M* ± *SD*)	3.58 ± 0.64	3.58 ± 0.47	3.17 ± 0.7	0.01*	0.02	0.02	0.99
Sense of security (*M* ± *SD*)	3.35 ± 0.46	3.13 ± 0.47	3.02 ± 0.45	0.01*	0.01	0.59	0.14
Participation (*M* ± *SD*)	3.24 ± 0.7	3.17 ± 0.71	2.62 ± 0.81	0.002*	0.003	0.01	0.92

*One-way ANOVA

**Tukey’s post hoc test

## Discussion

Regarding the study objective, comparison of the three groups after the intervention showed a statistically significant difference in the mean score of childbirth experience among the three groups. Accordingly, the primiparous women who used the two methods of delivery balls and warm showers had a better childbirth experience than the control group. Non-pharmacological interventions, such as the use of delivery balls, promote women’s mental health, help them better understand their active role during labor care, and stimulate a sense of participation in them [24]. The full presence of the midwife with the mother during ball exercises strengthens the mother’s sense of support and security [15]. In addition, the presence of a therapist in direct contact with pregnant women looks like the presence of a companion with the same beneficial effects, which causes them to be satisfied with childbirth [20]. In addition, it shows the positive effect of support during delivery on the childbirth experience, being in line with the present study.

In the study of Lathrap et al, mothers who experienced a water birth obtained significantly higher scores of childbirth experience, individual ability, and participation, yet no difference was reported in the fields of the sense of security and professional support [25]. The overall score of childbirth experience in the mentioned study was consistent with that of our study, but the results differed in some fields. Differences in the sample size, especially in the number of primiparous women and intervention methods, could be the reason for different results in the two studies.

Another study in Sweden examined the effect of the water birth on the childbirth experience in nulliparous and multiparous women. Accordingly, it concluded that the scores of childbirth experience and the sense of security were higher in nulliparous women sitting in water during the first stage of labor using a tube in a free position, who got out of the water at the beginning of the second stage. However, the increase in scores was not statistically significant. In addition, the score of the individual ability was significantly higher, yet the scores of participation and professional support were lower in nulliparous women than in multiparous ones, although not statistically significant [26]. The results of this study were different from those of the present study. This could be due to the fact that these women felt to be less in need of professional support than primiparous ones in the conventional (control) delivery group, due to their high individual ability. In addition, the childbirth experience in the above study was 6 weeks after delivery. Thus, according to the study of Turkmen et al (2018), the scores of participation and professional support significantly decreased after 3 months of childbirth [26].

Given the long-term effects of childbirth experience on women’s physical and mental health and taking into account the Oxford Summit’s emphasis on the prevention of psychological birth trauma (PBT) that negatively affects childbirth experience [27], studies and interventions aimed to create a positive childbirth experience are considered effective alternatives and solutions [10, 28]. In using warm showers, the effect of heat on the sacrum region leads to a reduction in pain intensity as well as the feelings of pleasure and comfort in women, through various mechanisms [29, 30]. On the other hand, hydrotherapy reduces the use of drugs and interventions with side effects such as fever [31], infections caused by bladder catheterization [32], delivery with spinal anesthesia [33], and uterine tachysystole [34]. Thus, hydrotherapy leads to normal physiological labor and delivery. Studies verify the safety of hydrotherapy for the mother, the fetus, and the infant. The use of warm showers, as a way of using humid heat, has the same benefits as hydrotherapy and heat therapy, which reduces labor pain and makes labor easier, thereby creating the feelings of comfort, security, support, and participation. In addition to the aforementioned benefits of delivery balls, warm showers create a positive childbirth experience in primiparous women.

### Strengths

The major strength of the present study was its controlled randomized design, which made it possible to conclude that the high birth experience score was affected by the interventions. Another strength of this study was the use of simulated squatting movements for working with the ball, while the squatting position was used in other studies, with no other similar study found on this issue. As far as we are concerned, no study has been conducted so far on comparing the two methods of delivery balls and hot showers, with their effects examined on childbirth experience.

### Limitations and future research

One of the limitations of present study was the conditions created by the COVID-19 pandemic at the time of the study, being associated with fear and anxiety about childbirth in women. In addition, the closure of childbirth preparation classes made it difficult for women to achieve required knowledge and information, especially about the pain during labor. In addition, single- or double-blinding was impossible due to the nature of the study; therefore, we attempted to mitigate this effect by randomly dividing the individuals into three groups. Given that the effect of midwife-centered interventions on multiparous women was not investigated in the present study, it is recommended that in future studies multiparous women be examined.

## Conclusion

The results of present study showed that the mean scores of childbirth experience and the fields of professional support and participation were significantly higher in both intervention groups than in the control group. In addition, the score of the sense of security was significantly higher in group A than in group C. It is recommended to use both interventions during delivery to achieve a more positive childbirth experience in women.

## Data Availability

Upon reasonable request of the corresponding author, qualified investigators will have access to the dataset under a Data Use Agreement.
